# Radiation-Associated Angiosarcoma of the Breast: A Case Report with Review of Reported Cases in Japan

**DOI:** 10.70352/scrj.cr.25-0777

**Published:** 2026-04-09

**Authors:** Eri Ida, Mahiro Ohashi, Fuminao Kanehisa, Momoko Komai, Naoko Itoi, Yoji Urata, Makoto Nagata, Tecchu Lee

**Affiliations:** 1 Department of Breast Surgery, Japanese Red Cross Kyoto Daiichi Hospital, Kyoto, Kyoto, Japan; 2 Department of Dermatology, Japanese Red Cross Kyoto Daiichi Hospital, Kyoto, Kyoto, Japan; 3 Department of Diagnostic Pathology, Japanese Red Cross Kyoto Daiichi Hospital, Kyoto, Kyoto, Japan

**Keywords:** angiosarcoma, breast cancer, mastectomy, radiotherapy, chemotherapy

## Abstract

**INTRODUCTION:**

Radiation-associated angiosarcoma of the breast (RAASB) is an extremely rare but serious complication that can occur several years after breast-conserving surgery and adjuvant radiotherapy. Owing to its rarity and nonspecific cutaneous manifestations, the diagnosis of RAASB is often delayed. There is no established treatment for RAASB, and its prognosis remains poor.

**CASE PRESENTATION:**

A 63-year-old woman developed progressive breast edema 14 years after breast-conserving surgery with axillary lymph node dissection and adjuvant radiotherapy for invasive ductal carcinoma. Two years later, she presented with breast masses with purpura and biopsy-confirmed angiosarcoma. Wide mastectomy with skin grafting was performed, followed by weekly administration of adjuvant paclitaxel. The patient remained recurrence-free for 12 months postoperatively.

**CONCLUSIONS:**

RAASB can develop long after breast-conserving therapy and may be preceded by subtle skin changes or persistent breast edema. Long-term follow-up and patient education are essential for patients who have undergone breast irradiation. Early imaging or biopsy should be considered when breast lymphedema is observed.

## Abbreviations

IDC
: invasive ductal carcinoma
MMG
: mammography
RAASB
: radiation-associated angiosarcoma of the breast
US
: ultrasonography

## INTRODUCTION

Angiosarcoma is a rare malignant tumor that accounts for less than 1% of all sarcomas. Angiosarcomas have also been reported to occur mainly in the skin (49.6%), followed by the breast (14.4%).^1)^ Breast angiosarcoma is extremely uncommon, accounting for approximately 0.04% of all malignant breast tumors. Owing to its rarity, an optimal treatment strategy has not yet been established. The clinical presentation is often nonspecific, and diagnosis can be delayed unless angiosarcoma is actively suspected. We encountered a case of RAASB that developed 16 years after breast-conserving surgery and reported this case with a brief review of the literature.

## CASE PRESENTATION

A 63-year-old woman underwent breast-conserving surgery with axillary lymph node dissection (level I) for IDC of the left breast (area C). The pathological diagnosis was IDC, pT1bN0M0, stage I, ER(+), PgR(+), HER2(−), and postoperative radiotherapy (50 Gy in 25 fractions) to the residual breast, followed by 5 years of adjuvant endocrine therapy. The patient was regularly followed up thereafter.

**Fig. 1:**
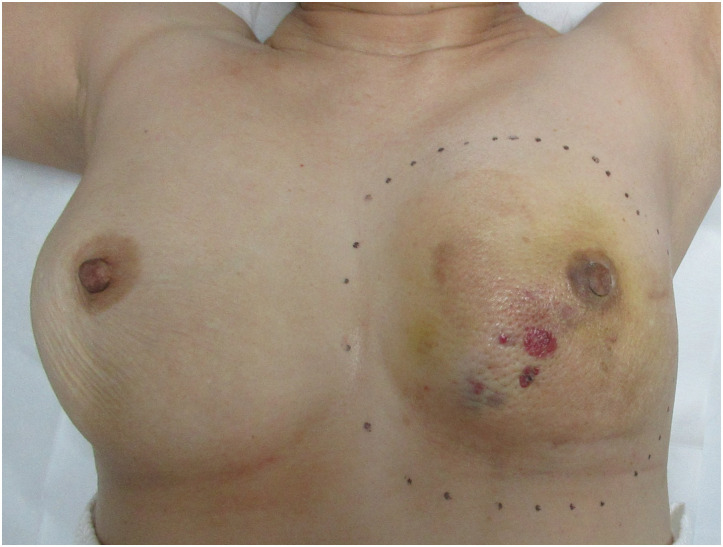
Macroscopic findings before surgery: Localized masses with purpura were observed in the left breast. The dashed line indicates the planned surgical resection margin.

**Fig. 2:**
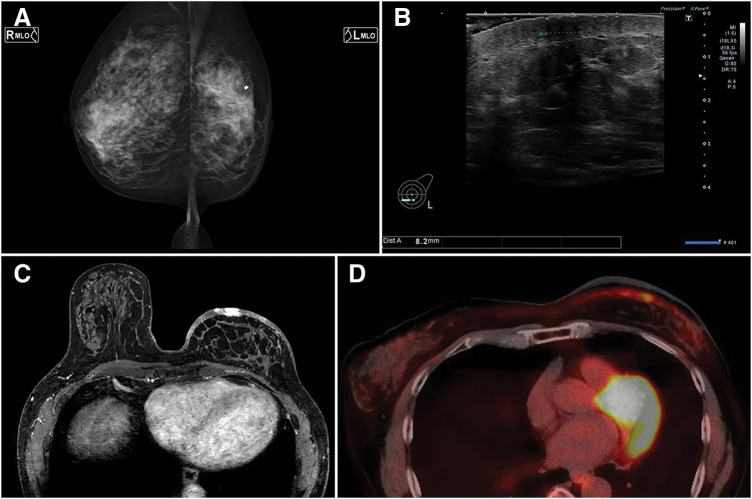
Imaging findings: (**A**) Mammography, (**B**) breast ultrasonography, (**C**) contrast-enhanced MRI, and (**D**) PET-CT.

**Fig. 3:**
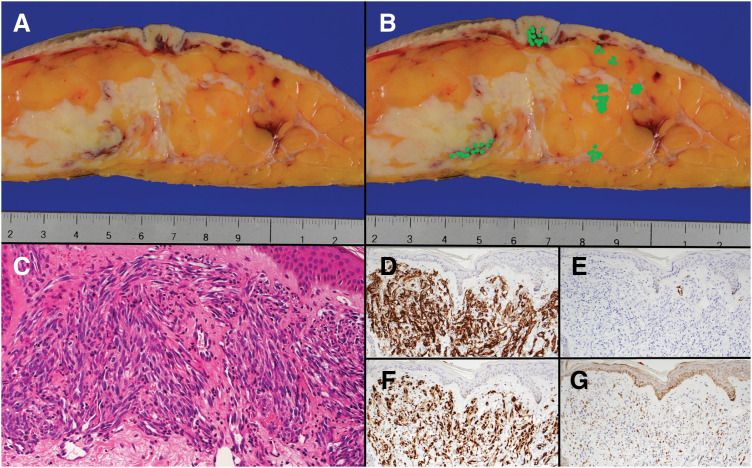
Gross cross-sectional view of the surgical specimen: (**A**) Original gross image, (**B**) the same image with c-Myc-positive areas marked by green dots. Histopathological and immunohistochemical findings: (**C**) Hematoxylin and eosin (H&E) staining, (**D**) CD31 positive, (**E**) D2-40 negative, (**F**) ERG positive, (**G**) c-myc positive.

Fourteen years after the surgery, she developed a swelling in the left breast. MMG and breast US revealed no obvious malignancy, and the condition was interpreted as breast lymphedema. Sixteen years after surgery, the patient presented with breast skin masses and purpura in the residual breast tissue (**Fig. 1**). MMG revealed postoperative changes and coarse calcifications in the left M-O area with associated skin edema. The right breast was categorized as category 1, and the left breast as category 2. In contrast, US revealed diffuse edema of the breast skin and a scar in area C, with no axillary lymphadenopathy. Contrast-enhanced MRI demonstrated skin thickening with early enhancement in the lower inner quadrant. PET-CT revealed increased uptake (maximum standardized uptake value [SUVmax] 4.6) beneath the left nipple without evidence of nodal or distant metastasis (**Fig. 2**). Punch biopsy of the violaceous lesion confirmed angiosarcoma. Immunohistochemical analysis revealed that the neoplastic cells were ERG (+), CD31 (+), CD34 (+), D2-40 (−), c-myc (+), AE1/AE3 (−), and had a Ki-67 index of approximately 70% (**Fig. 3**).

**Fig. 4:**
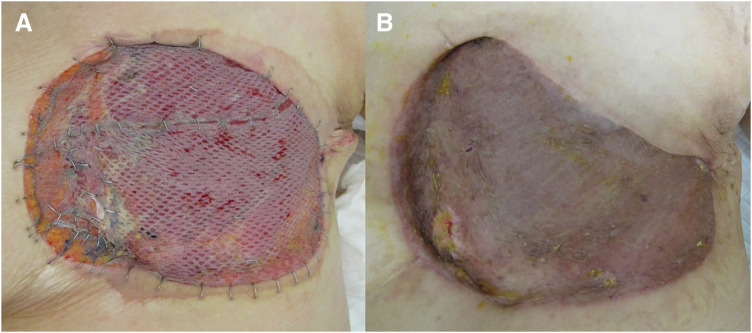
Macroscopic findings: (**A**) One week after surgery. (**B**) One month after surgery.

A wide mastectomy was performed with a 2-cm margin beyond the visible purpura, including the superficial fascia of the pectoralis major (**Fig. 1**). Six margin samples were subjected to intraoperative frozen section analysis, all of which were negative. The skin defect was reconstructed using a split-thickness skin graft harvested from the right thigh (meshed and stapled) (**Fig. 4**).

Gross pathological examination revealed a 10 × 10 × 5-cm mass. Histopathologically, the mass was composed of thin-walled, anastomosing vascular channels lined by atypical endothelial cells with nuclear enlargement, as well as solid areas of short-spindle cells. Immunostaining demonstrated c-myc overexpression, and all surgical margins were negative for c-myc-positive vessels (**Fig. 3**).

The postoperative course was uneventful. Weekly adjuvant paclitaxel (PTX; 80 mg/body) was initiated, and 15 cycles (3 consecutive weeks followed by 1 week off) had been administered at the time of reporting. The patient remained recurrence-free for 12 months postoperatively.

## DISCUSSION

Breast-conserving surgery followed by radiotherapy is the standard treatment for early-stage breast cancer.^2)^ However, RAASB is a rare complication that occurs late. Its pathogenesis has been attributed to (1) chronic lymphedema following axillary dissection and (2) radiation-induced DNA damage, leading to the accumulation of repair errors and chromosomal instability.^3–5)^

We reviewed 40 Japanese cases of RAASB reported between 2014 and 2024, involving 41 patients (**Table 1** and **Supplementary Table 1**).^6–37)^ The median age at diagnosis and latency period after radiotherapy in Japanese patients were comparable to those reported in Western cohorts. However, in Western series, RAASB is typically diagnosed only after the appearance of overt cutaneous lesions or nodular tumors of considerable size.^38)^ In contrast, several Japanese cases, including the present case, suggest that subtle skin changes or persistent breast edema may precede tumor detection. This difference may indicate a potential window for earlier clinical suspicion in patients with a history of breast irradiation.

**Table 1:** Summary of reported Japanese cases of radiation-associated angiosarcoma of the breast

	Median [range]
Age	74 [32–95]
Latency after radiation	n (%)
≤5 years	12 (29)
>5–10 years	20 (49)
>10 years	9 (22)
Symptoms	n (%)
Skin changes	30 (73)
Mass or nodule	21 (51)
Pain or pruritus	6 (15)
Type of surgery	n (%)
Mastectomy	31 (76)
Partial resection	7 (17)
Not described	3 (7)
Skin grafting	18 (44)
Chemotherapy regimen*	n (%)
PTX or nab-PTX	21 (51)
Pazopanib	2 (5)
Trabectedin	2 (5)
Docetaxel	2 (5)
Eribulin	2 (5)
Teceleukin	1 (2)
Bevacizumab	1 (2)
Recurrence	n (%)
Any recurrence	20 (49)
Local recurrence	14 (34)
Distant metastases**	10 (24)
Liver	5
Bone	3
Lung	2
Pleura	2
Lymph node	2
Knee joint	1
Time to recurrence	n (%)
≤6 months	10 (24)
>6–12 months	6 (15)
>12 months	4 (10)

*Some patients received multiple regimens.

**Some patients had multiple metastatic sites.

nab-PTX, nanoparticle albumin-bound paclitaxel; PTX, paclitaxel

One possible explanation for this earlier phase is the presence of chronic lymphatic disturbance within irradiated breast tissue. Chronic lymphatic stasis is known to generate a pro-tumorigenic microenvironment characterized by persistent inflammation, tissue hypoxia, and impaired immune surveillance, as exemplified by Stewart–Treves syndrome of the upper extremity. In the present case, although the anatomical site differs, a similar pathophysiological process may have occurred within the breast. Thus, in addition to radiation-induced DNA damage, chronic lymphatic disturbance and the resulting microenvironmental alterations may have acted synergistically to promote the development of secondary angiosarcoma.

In our case, RAASB developed 16 years after surgery. In the present review, 10 cases were reported to have developed RAASB more than 10 years after the initial treatment. These findings suggest that long-term follow-up is required for patients with breast cancer who have undergone breast irradiation. However, in routine clinical practice, postoperative follow-up is often completed long before the onset of RAASB. Therefore, patient education is essential, and patients should be encouraged to seek medical attention if they notice breast edema. In addition, when breast lymphedema is identified in patients with a history of breast irradiation during breast cancer screening or evaluation for other medical conditions, clinicians should remain alert to the possibility of RAASB.

Histologically, RAASB is characterized by positivity for endothelial markers and overexpression of c-myc, a hallmark of radiation-induced angiosarcoma.^13,39)^ In the present case, c-myc overexpression supported the diagnosis and was useful for delineating the extent of the tumor. In previously reported Japanese cases, CD31 and CD34 immunostaining was performed in most patients and showed positivity in more than 90% of cases, indicating their widespread use as reliable diagnostic endothelial markers. However, unlike conventional endothelial markers that simply confirm differentiation, c-myc overexpression is highly specific for radiation-associated angiosarcoma and provides additional diagnostic and biological relevance. Other markers such as D2-40 and S-100 were also variably examined, mainly excluding lymphatic or melanocytic differentiation.

According to the 2025 Japanese Dermatological Association guidelines for cutaneous angiosarcoma, surgical resection is the first-line treatment, although optimal margins have not yet been established.^40)^ Margins ≤1 cm are associated with poor prognosis, yet recurrence may occur even after wide margins. In our case, a 2-cm margin was confirmed intraoperatively with frozen sections. International guidelines also emphasize complete resection with negative margins, although the recommended margin width varies across regions.^41,42)^ In our review of RAASB, mastectomy was performed in 31 of 41 patients (76%), and 18 patients (44%) required skin grafting.

PTX is considered a first-line chemotherapeutic agent for angiosarcoma in Japan, along with other approved options such as pazopanib, trabectedin, and eribulin.^40)^ Among the 41 reviewed cases, 22 (54%) received chemotherapy, and 21 (95%) received PTX or nab-PTX as first-line therapy (**Supplementary Table 1**). In previous Japanese reports of RAASB, the median number of PTX cycles was 12 (range, 6–50), with a median treatment duration of approximately 12 months after surgery and a median disease-free survival of 19 months.^43)^ However, the optimal duration of chemotherapy remains unclear. At our institution, patients with angiosarcoma are generally managed as follows. 1) Chemotherapy is initiated with PTX or nab-PTX as first-line treatment. 2) If the patient remains tumor-free and tolerates treatment well, chemotherapy is continued for approximately 1–2 years after surgery, following discussion with the patient. 3) If adverse events such as peripheral neuropathy make continued administration difficult, treatment is discontinued and the patient is carefully observed once more than 12 months have elapsed after surgery. 4) If treatment discontinuation is required earlier than 12 months postoperatively, alternative agents approved for soft tissue sarcoma in Japan, such as eribulin or pazopanib, are considered. In this case, chemotherapy is continued postoperatively as long as the patient maintains a good performance score.

Although the patient has remained recurrence-free for 12 months after surgery, RAASB is known to have a high risk of local and distant recurrence. In our review of 41 Japanese cases, time to recurrence was reported in 20 cases, with a median of only 7 months (range, 1–36 months). Moreover, the prognosis of RAASB remains poor, with a reported 5-year disease-free survival rate of approximately 34%.^6)^ Accordingly, careful long-term follow-up is required to fully assess the oncologic outcome in this patient.

## CONCLUSIONS

In patients with breast cancer who have undergone breast irradiation, long-term follow-up and patient education are essential because of the potential risk of RAASB. When breast lymphedema is present, closer surveillance is recommended, and earlier imaging or biopsy should be considered.

## SUPPLEMENTARY MATERIALS

Supplementary Table 1

## DECLARATIONS

### Availability of data and materials

All data generated or analyzed during this study are included in this article.
