# Supplementary material for: Radiation-Associated Angiosarcoma of the Breast: A Case Report with Review of Reported Cases in Japan
Source: Surg Case Rep. 2026 Apr 9;12(1):25-0777. doi: 10.70352/scrj.cr.25-0777 (PMC13070623; doi:10.70352/scrj.cr.25-0777)
Supplement: Supplementary Table 1 [file scr-12-01-25-0777-s001.pdf]

Supplementary Table 1

Publication year	Author	Age	Time since surgery (year)	Tumor size (cm)	Symptoms	Type of surgery	Adjuvant treatment	Organ of recurrence	Time to recurrence (months)
2014	Kimura	69	5	3.4	Induration	Bp	–	–	—
		61	11	10	Erythema, Induration	Bt + Skin graft	PTX	–	–
2014	Yasuoka	89	6	1	Intramammary hemorrhage, mass	Bp	DTX	Local	2
2014	Todoroki	53	7	2.4	Skin thickening, Subcutaneous hemorrhage	Bt + Skin graft	–	Local	4
2014	Mizumoto	78	4	16	Erythema, Induration, Erosion	Bt + Ax	–	Pleura, Knee joint	12
2014	Hojo	64	7	0.7	Mass	Bp	–	Local	4
2014	Ogata	32	6	5.6	Mass, Subcutaneous hemorrhage	Bt + Skin graft	PTX	Liver, Bone, Local	12
2014	Tajima	73	5	Unknown	Mass, Pigmentation, Crust	Bt	PTX	–	—
2015	Oda	80	13	1.2	Induration, Purpura	Bp	Unknown	Unknown	—
2016	Hara	81	10	Unknown	Induration, Pain	Bp	nab-PTX	Pleura	5
2016	Kobayashi	82	13	Unknown	Hemorrhage, Mass	RM	–	Local	13
2016	Masai	74	7	26	Mass	CWR	–	Liver	11
2017	Kanaizumi	77	6	1	Purpura, Nodule	Bt + Skin graft	PTX	Liver	19
		53	4	4	Erythema, Purpura	Bt + Skin graft	–	–	—
2017	Kawasaki	81	4	5.5	Mass	Bt + Skin graft	PTX	Local	3
2017	Sawatsubashi	82	7	Unknown	Mass	Bt	–	Local	1
2018	Takamatsu	77	6	4.5	Skin thickening, Purpura	Bt + Skin graft	PTX	–	—
2018	Horisawa	76	9	0.6	Nodule, Pigmentation	Wide skin excision + Skin graft	–	–	—
2018	Nomoto	64	4	4.5	Nodule, Skin thickening	Bt + Skin graft	PTX	–	—
		67	3	Unknown	Nodule	Bt + Skin graft	nab-PTX	–	—
2018	Tokoro	74	14	10	Nodule	Bt+Ax	PTX	Bone, Lung, Liver	8
		76	7	7	Poor granulation, Bloody nipple discharge	Bp	PTX	–	—
2020	Tamaoki	72	5	13	Hemorrhage blister	Bt + LD flap reconstruction	–	Local	6
2020	Takada	86	7	3.5	Hemorrhage, Mass	Bt	PTX+Bev	Local	1
		67	6	Unknown	Reddish-purple rash	Bt + Skin graft	–	–	—
2020	Miyata	88	7	Unknown	Erythema, Mass	None	PTX	–	—
2020	Takatsuka	76	7	7.1	Nodule	Bt + Skin graft	–	Local, Lymph node	22
2020	Suzuki	62	8	3	Nodule	Bt	PTX	Local	1
2020	Shiraki	72	5	18	Erythema, Nipple enlargement	Bt + Skin graft	PTX, TRB	Local	12
2020	Mizusaki	82	4	9	Nodule, Yellowish patch	Bt + Skin graft	–	–	—
2020	Ishizuka	81	9	1.7	Dark purplish nodule, Erythema	Bt + Skin graft	–	–	—
2022	Iijima	74	4	Unknown	Skin lesion, Induration, Purpura	Bt	–	–	—
2022	Yonekawa	76	7	1.8	Induration	Bp	–	–	—
2022	Hojo	58	6	10.2	Breast edema	Bt	PTX, Pazopanib, ERI, TRB	Lung, Local	3
2023	Watanabe	66	14	16	Erythema, Pain, Swelling	Bt + Skin graft	nab-PTX	–	—
		67	11	18	Subcutaneous hemorrhage	None	PTX, Pazopanib, ERI, DTX + Teceleukin	Lymph node	36
2023	Saito	80	15	3.4	Mass	Bt	–	–	—
2023	Ono	75	7	0.5	Nodule	Bt + Skin graft	PTX	Liver, Bone, Local	12
2024	Yamada	95	11	Unknown	Nipple erythema, Pain, Nipple discharge	Bt	–	Unknown	—
		61	5	Unknown	Itching, Induration	Bt	PTX	–	—
2025	Our case	63	16	10	Swelling, Subcutaneous hemorrhage, Mass	Bt + Skin graft	PTX	–	—

Abbreviations: DFS; disease free survival, Bt; Breast total resection, Bp; Breast partial resection, Ax; Axillary lymph node dissection, RM; Radical mastectomy, CWR; mastectomy with chest wall resection, PTX; paclitaxel, DTX; docetaxel, ERI; eribulin, TRB; trabectedin, Bev; bevacizumab, LD; Latissimus dorsi
